# Homozygous PPP1R13L Mutation Associated with Dilated Cardiomyopathy in a 1-Year-Old Child

**DOI:** 10.3390/life16081229

**Published:** 2026-07-24

**Authors:** Adelina-Mihaela Sorescu, Cristina Isabel Viorica Ghiță, Gabriela Duică, Alin Nicolescu, Eliza Elena Cinteză, Rachele Adorisio, Antonio Amodeo, Paola Francalanci, Gianluca Brancaccio, Gessica Ingrasciotta, Erica Mencarelli, Oana Andreia Coman

**Affiliations:** 1 Department I—Functional Sciences, Pharmacology, Clinical Pharmacology and Pharmacotherapy, “Carol Davila” University of Medicine and Pharmacy, 050474 Bucharest, Romania; adelina-mihaela.enache@drd.umfcd.ro (A.-M.S.); oana.coman@umfcd.ro (O.A.C.); 2 Pediatrics Department, “Carol Davila” University of Medicine and Pharmacy, 050474 Bucharest, Romania; elizacinteza@yahoo.com; 3 Marie S. Curie Clinical Emergency Hospital for Children, 077120 Bucharest, Romania; nicolescu_a@yahoo.com; 4 Heart Failure, Transplantation and Cardio-Respiratory Mechanical Assistance Unit, Bambino Gesù Children’s Hospital, IRCCS, Piazza Sant’Onofrio 4, 00165 Rome, Italy; rachele.adorisio@opbg.net (R.A.); antonio.amodeo@opbg.net (A.A.); gessica.ingrasciotta@opbg.net (G.I.); erica.mencarelli@opbg.net (E.M.); 5 Department of Cardiothoracic Surgery, Stanford University, Stanford, CA 94305, USA; 6 Pathology Unit, Bambino Gesù Children’s Hospital, IRCCS, Piazza Sant’Onofrio 4, 00165 Rome, Italy; paola.francalanci@opbg.net; 7 Cardiac Surgery Unit, Bambino Gesù Children’s Hospital, IRCCS, Piazza Sant’Onofrio 4, 00165 Rome, Italy; gianluca.brancaccio@opbg.net

**Keywords:** *PPP1R13L*, dilated cardiomyopathy, cardiocutaneous syndrome

## Abstract

**Introduction:** Dilated cardiomyopathy (DCM) is a relatively rare manifestation of pediatric heart failure. Despite recent diagnostic advancements and expanded screening modalities, the underlying cause of DCM remains elusive in over 50% of pediatric cases. We report the case of a 1-year-old girl presenting with newly diagnosed, rapidly progressive dilated cardiomyopathy, caused by a novel, rare homozygous pathogenic variant in the *PPP1R13L* gene. **Case presentation:** The patient, with no significant family history of cardiac disease, previously asymptomatic and in good health, presented with a 2-day history of nausea, vomiting, difficulty breathing, and low urine output, following a recent respiratory infection. The echocardiography identified a dilated left ventricle (Z score > +2 for age) and severe systolic left ventricular dysfunction, and a positive diagnosis of dilated cardiomyopathy was confirmed by the cardiac magnetic resonance (CMR). Genetic testing identified a homozygous pathogenic variant in the *PPP1R13L* gene, classified as a novel pathogenic variant that encodes the inhibitor of apoptosis-stimulating protein of p53 (iASPP). Despite targeted treatment, the severe systolic dysfunction persisted, and the general state progressively deteriorated and warranted the need for a cardiac transplant, which she underwent one year following the initial diagnosis. **Conclusions:** Patients diagnosed with underlying genetic DCM, particularly those harboring *PPP1R13L* mutations, carry an exceedingly poor prognosis in the absence of orthotopic heart transplantation. Existent case reports confirm that disruptions in this gene predictably yield highly aggressive, life-threatening cardiomyopathies.

## 1. Introduction

Pediatric heart failure (HF) arises from a diverse spectrum of etiologies, with cardiomyopathies increasingly recognized as primary drivers of cardiac dysfunction in recent years [1]. Although cardiomyopathies represent a relatively rare manifestation of pediatric heart failure—with an estimated annual incidence of 0.6 to 0.7 cases per 100,000 children—they impose a disproportionately high public health and economic burden due to the extensive resource utilization, prolonged hospitalizations, and specialized advanced therapies they necessitate [2,3].

Given its highly heterogeneous etiology, dilated cardiomyopathy (DCM) is broadly classified into primary forms, which are predominantly genetic, and secondary forms resulting from cardiac or systemic insults, including myocarditis, tachyarrhythmias, systemic infections, nutritional deficiencies, and toxic exposures [4,5,6,7,8,9,10,11,12]. Despite recent diagnostic advancements and expanded screening modalities, the underlying cause of DCM remains elusive in over 50% of pediatric cases, leaving a substantial proportion of patients categorized as having idiopathic dilated cardiomyopathy [13,14,15].

Clinically, DCM manifests as progressive heart failure with highly non-specific signs and symptoms. Depending on the patient’s age, disease trajectory, and primary etiology, presentation can range from subtle respiratory or gastrointestinal distress to acute, life-threatening cardiogenic shock [5,16,17,18]. Echocardiographically, the diagnostic hallmark relies on two essential criteria: left ventricular dilation, defined as a left ventricular end-diastolic diameter (LVEDD) exceeding two standard deviations (>2 SD) above the age-adjusted mean, and concurrent systolic dysfunction, characterized by an ejection fraction or fractional shortening falling more than two standard deviations (<2 SD) below normal reference values [4,19,20,21]. Management difficulties in pediatric DCM stem primarily from the absence of a definitive etiological diagnosis in the majority of cases. Beyond initial biomarker screening and routine laboratory investigations, multimodality imaging remains central to the diagnostic pathway. While transthoracic echocardiography is the most accessible tool for baseline evaluation, comprehensive metabolic profiling, molecular genetic testing, advanced imaging protocols—such as cardiac magnetic resonance (CMR) imaging—and endomyocardial biopsy are critical to uncovering the precise underlying molecular mechanisms and tailoring individual therapeutic strategies [16,22].

Although the landmark genetic and genotype–phenotype association studies in DCM have historically centered on adult cohorts, an increasing number of distinct pathogenic variants are being actively uncovered in pediatric patients [23]. The historical scarcity of pediatric-specific genomic data represents a critical clinical challenge; identifying a precise molecular correlate is vital, as it can radically alter targeted management regimens and facilitate informed familial reproductive counseling [24]. Recently, biallelic variants in the *PPP1R13L* gene have emerged as a novel genetic substrate driving aggressive early-onset cardiomyopathy [24,25]. *PPP1R13L* encodes the inhibitor of apoptosis-stimulating protein of p53 (iASPP), a critical protein that downregulates pro-inflammatory pathways and regulates desmosomal integrity within tissues exposed to high mechanical stress [25]. Defective iASPP signaling impairs the heart’s threshold to inflammatory stressors and compromises cardiomyocyte adhesion, culminating in extended phenotypic spectrums that include cardiocutaneous syndrome, severe dilated cardiomyopathy, and arrhythmogenic phenotypes.

Presently, standardized, evidence-based guidelines for pediatric DCM are lacking due to a scarcity of prospective clinical trials evaluating drug efficacy and safety profiles in children. Consequently, conventional pharmacotherapy for pediatric heart failure relies heavily on data extrapolated from large-scale clinical trials involving adult populations [26,27,28,29]. The clinical trajectory of pediatric DCM remains highly variable and unpredictable, typically following one of three paths: partial to complete recovery of ventricular function, long-term clinical stabilization under optimal medical therapy, or rapid, refractory hemodynamic deterioration necessitating orthotopic heart transplantation or culminating in mortality. Among the various predictors of long-term survival, the specific underlying etiology stands out as perhaps the most critical prognostic determinant.

In this context, we report the case of a 1-year-old girl presenting with newly diagnosed, rapidly progressive dilated cardiomyopathy. Molecular genetic testing revealed a rare homozygous pathogenic variant in the *PPP1R13L* gene. Reflecting the malignant, treatment-refractory trajectory typical of this genetic subset, the patient experienced accelerated clinical decline that proved unresponsive to conventional pharmacological interventions, ultimately requiring emergent orthotopic cardiac transplantation.

### 1.1. Case Presentation

The patient was referred to the pediatric cardiology department of the Clinical Emergency Hospital for Children “Marie S. Curie” from a private practice hospital. The patient presented with a 2-day history of nausea, vomiting, difficulty breathing, and low urine output. Initial chest radiography revealed cardiomegaly, while echocardiography demonstrated severe systolic dysfunction. Consequently, the patient was transferred to our hospital for further investigations and management.

The patient had no significant family history of cardiac disease; she was the first child, born at a normal gestational age, with a birth weight of 2400 g, an Apgar score of nine, with good postnatal adaptation and good psychomotor development over the years. Prior to this presentation, the patient had been asymptomatic and in good health, with the exception of several respiratory infections, the most recent one being one month prior to presentation.

### 1.2. Clinical Findings on Admission

Following admission, the patient was in a relatively good general state, afebrile, with no infectious symptoms. At admission, with a weight of 10 kg and a height of 110 cm, she presented weight-for-age and height-for-age below normal standards. The physical examination revealed pale, warm, dry skin, with no desquamation, brittle and friable nails, and short, fine, thin, and sparse hair. The respiratory exam showed an increased respiratory rate of 60 rpm, with dyspnea and moderate respiratory distress, 90% oxygenation with no oxygen supplementation, and normal breath sounds. The cardiac exam was characterized by tachycardia (heart rate = 150 bpm), normal blood pressure (BP = 110/80 mmHg), normal peripheral pulses, a mild systolic murmur, and periorbital edema. She also presented with hepatomegaly and low urine output.

### 1.3. Investigations

Paraclinical investigations were determined to assess the severity and the systemic impact of the disease, as well as identify a possible underlying etiology.

Laboratory investigations revealed moderate normocytic, normochromic anemia, with a hemoglobin level of 9 g/dL (normal range: 9.4–13 g/dL), with a normal leukocyte formula. No biological inflammatory syndrome was identified. Hepatic and renal function tests were within normal limits. Regarding the cardiac biomarkers, the creatinkinase level was 131 U/L (normal range: 7–295 U/L), but with high levels of 32.5 U/L creatinkinase fraction MB (normal range: 7–25 U/L), while the troponin levels were elevated (1896 ng/L) (normal range: <40 ng/L), as well as the NT-proBNP levels (50,000 pg/mL) (normal range: <125 pg/mL). D-dimers were positive (2.03 mcg/mL) (normal range 0–0.5 mcg/mL), while the coagulation tests remained normal. Viral serologies were tested, but only revealed a high level of IgG antibodies (20.6 U/mL) (normal range < 0.5 U/mL) for Cytomegalovirus.

**Figure 1:**
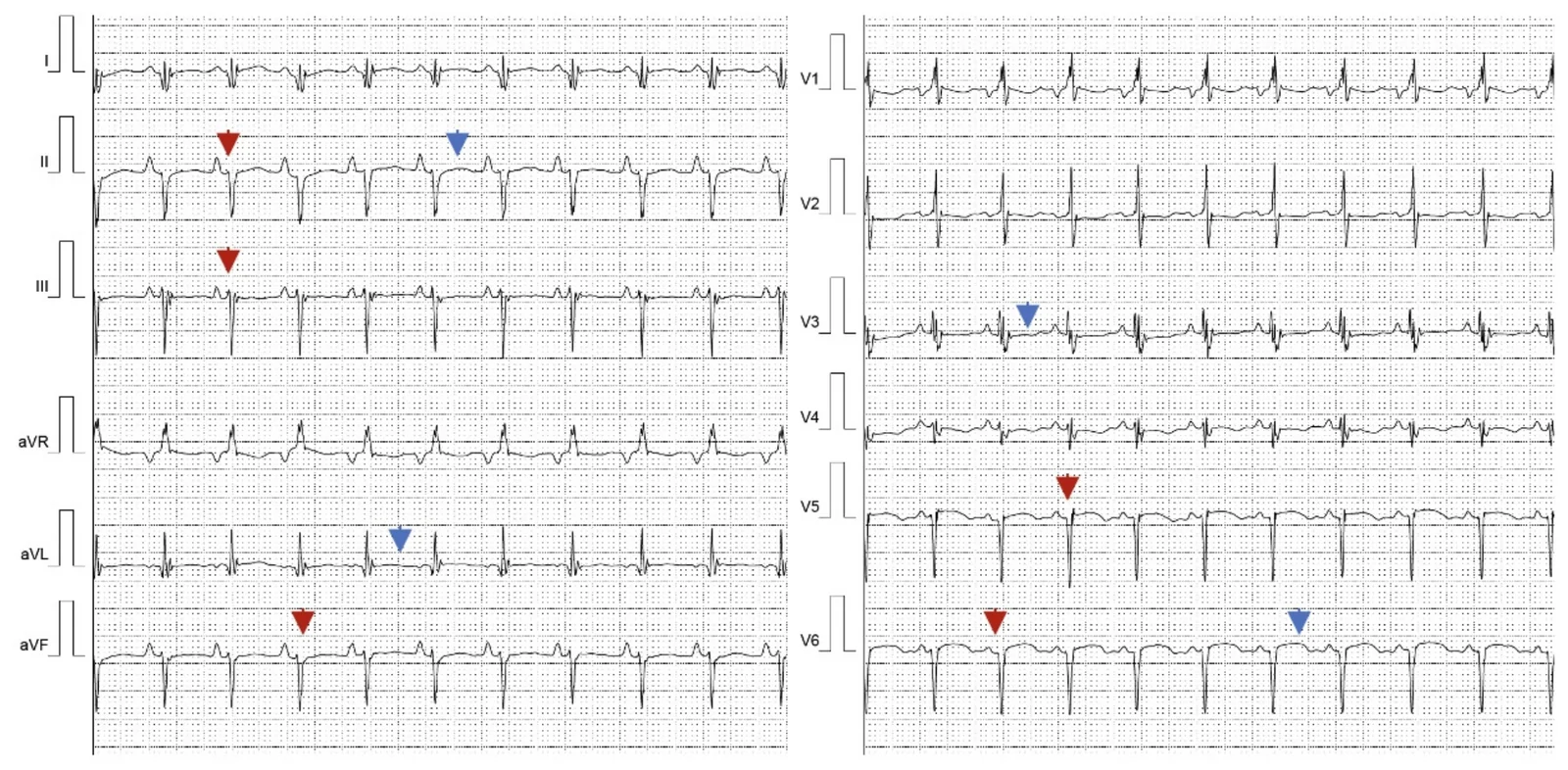
Electrocardiographic finding showing a leftward ventricular axis, Q waves present in the inferior and lateral leads (red arrows), and diffuse T-wave flattening (blue arrows).

A sinus rhythm was seen on the electrocardiogram (ECG), with normal P waves, a PR interval of 120 ms, a leftward ventricular axis, and a QRS duration of 60 ms. Q waves were seen in the inferior leads and V4-V6 leads, with diffuse T-wave flattening (Figure 1).

The continuous ECG Holter monitoring showed isolated monomorphic ventricular premature beats, with no other arrhythmic events and no other conduction disturbances.

The main imaging exam used was the echocardiogram which made a positive diagnosis of dilated cardiomyopathy, with a dilated left ventricle (Z score > +2 for age), severe systolic left ventricular dysfunction (ejection fraction (EF) of 11% calculated with the Simpson method), aortic VTI of 139 mm, pulmonary VTI of 80 mm, left ventricular TEI index of 0.56 and a calculated cardiac output of 1.6 L/min. Regarding the right ventricle, it presented with a moderate systolic dysfunction (ejection fraction of 35%, calculated with the help of the fractional area change). The coronary arteries had normal origins and dimensions, and no other structural anomalies were seen. The patient also had mild mitral regurgitation and moderate tricuspid regurgitation. The inferior vena cava and the suprahepatic veins were dilated, with partial respiratory collapse. The patient presented with small pericardial effusions and small bilateral pleural effusions.

**Figure 2:**
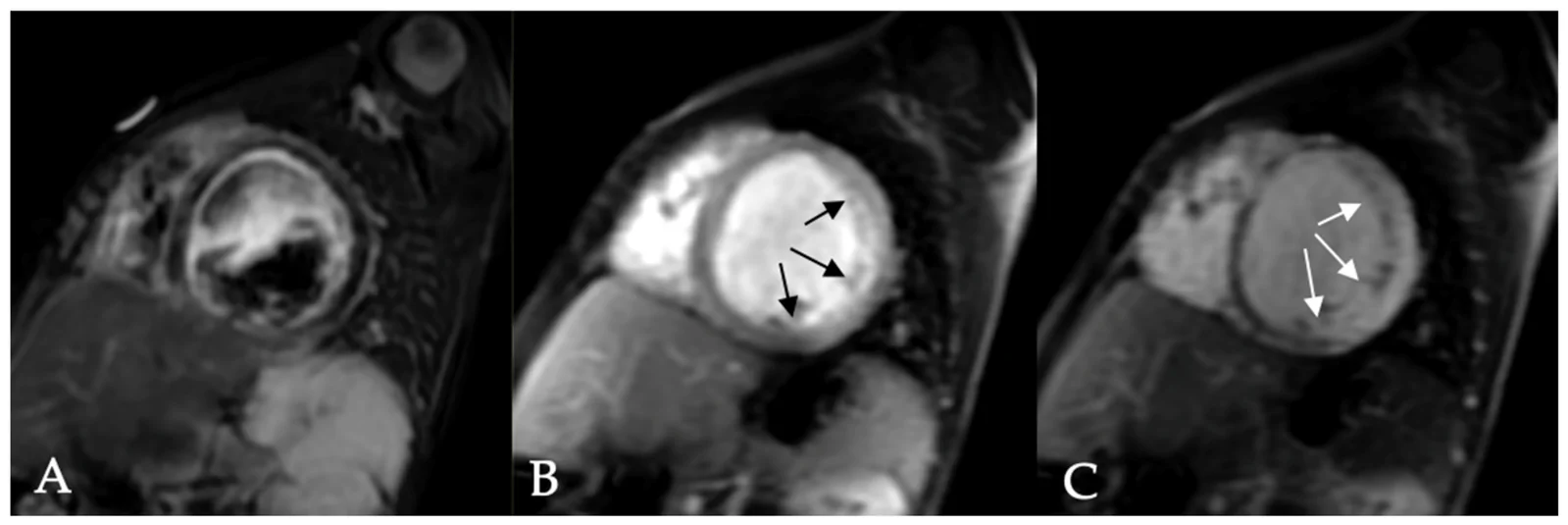
Cardiac magnetic resonance showing diffuse chamber dilation and thin left ventricular walls. Image (**A**)—T2 fs image showing no inflammation; Image (**B**)—EGE image showing early transmural contrast enhancement of the free ventricular wall (black arrows); Image (**C**)—LGE image showing late transmural contrast enhancement of the free left ventricular wall (white arrows). EGE, early gadolinium enhancement; LGE, late gadolinium enhancement.

Given the clinical presentation, the echocardiographic aspect, and the recent history of infectious disease, a diagnosis of acute myocarditis was taken into consideration. For this reason, and in order to better investigate the myocardial structure, a cardiac magnetic resonance (CMR) was performed. The CMR was performed on a 1.5 T MRI General Electric Signa Explorer. The findings on this investigation were consistent with the echocardiographical aspect, as a severe dilation of the left ventricle (end diastolic volume of 180 mL/m^2^) and important biventricular systolic dysfunction (left ventricular EF of 5% and right ventricular EF of 14%) were seen. The CMR also revealed a diffuse transmural contrast enhancement of the left ventricular wall on both early and late imaging sequences (Figure 2). The findings were suggestive of a possible inflammatory myocardial injury superimposed on a chronic cardiomyopathy. Despite the presence of early gadolinium enhancement on the T2 mapping CMR images, the clinical and paraclinical criteria (including the Lake Louise criteria) for diagnosing an acute myocarditis were not met. However, due to technical issues, this diagnosis could not be either confirmed or refuted, as an endomyocardial biopsy could not be performed at that time in our clinic.

However, the patient had previously been asymptomatic, in spite of the severe dilation and systolic dysfunction of the left ventricle. This was explained by a potential preexisting chronic dilated cardiomyopathy. That is why we continued the investigations in order to determine an etiological diagnosis of the disease.

Genetic testing identified a homozygous pathogenic variant in the *PPP1R13L* gene, Exon 12, (NM_006663.4:c.2257C>T, p.(Gln753*)), classified as a novel pathogenic variant that encodes iASPP. This is a single-base substitution, replacing Glutamine with a Termination codon in the *PPP1R13L* gene. Truncated variants in the *PPP1R13L* gene are known to be pathogenic. Based on the classification criteria set by the ACMG and AMP, this variant has been classified as pathogenic. The variant can cause arrhythmogenic cardiomyopathy and various ectodermal abnormalities. It is transmitted in an autosomal recessive manner.

During the genetic testing, sequencing was carried out using the DNBSEQ-G400 sequencing platform (MGI). Reads were aligned to the reference (GRCh37), and sequence changes were identified in the context of a single clinically relevant transcript. Analysis of the mutations with damaging effect (frameshift, nonsense, missense, splicing mutations, etc.) as well as de novo mutations was obtained. All clinically significant observations were confirmed by Sanger Sequencing analysis on SeqStudio Genetic Analyzer (Thermo Fisher Scientific). The presence of large genomic rearrangements (LGRs) was investigated using the commercial computational algorithm SeqPilot Version 4.4 Build 505 (JSI Medical System).

In order to further investigate the population prevalence and the genetic transmission, genetic testing of the parents was conducted one month ago, and the results are still in progress.

Metabolic testing was negative and showed no abnormalities, while the nutritional status of the patient was characterized by lower levels of carnitine and leucine.

No dermatological consult was performed during admission.

### 1.4. Treatment and Follow-Up

Following the diagnosis, the patient received heart failure treatment, including a loop diuretic, (Furosemide initially administered intravenously for approximately one week and continued orally during admission and upon discharge and angiotensin converting enzyme (ACE) inhibitor (Lisinopril, oral, 0.1 mg/kg/day), aldosterone receptor blocker (Spironolactone, oral, 1 mg/kg/day), and beta blocker (Bisoprolol, oral 0.1 mg/kg/day). Given the systolic dysfunction and the chamber dilation, antithrombotic medication was administered, consisting initially of subcutaneous Enoxaparin for one week, followed by oral Aspirin upon discharge.

In the context of the acute heart failure symptomatology, without a biological inflammatory syndrome, but with severe systolic dysfunction and the CMR aspect of early and late gadolinium enhancement, intravenous immunoglobulin was considered in this patient, even though acute myocarditis was not confirmed. Two doses of 2 g/kg of intravenous immunoglobulin were administered. Apart from this, supplemental carnitine and leucine were administered in the nutritional context.

The patient presented a favorable clinical and paraclinical evolution under targeted therapy. Following intensive diuretic treatment, she achieved significant symptomatic improvement, with minimal respiratory effort, resolution of peripheral edema, and complete disappearance of both pleural and pericardial effusions.

Serial echocardiographic assessments revealed a gradual but slow improvement in global ventricular function, with the left ventricular ejection fraction increasing from 11% at presentation to 20% during hospitalization. Cardiac biomarkers also showed a downward trend: NT-proBNP levels decreased to 19,590 pg/mL, while troponin levels declined from 1896 ng/L to 1082 ng/L.

At discharge, the patient was asymptomatic. Despite further improvement in ventricular function, with both left and right ventricular ejection fractions reaching 25%, echocardiography continued to demonstrate severe biventricular systolic dysfunction. Although cardiac biomarker levels were substantially lower at discharge than at admission, they remained above the normal range.

Approximately two months after the initial diagnosis, the patient experienced a new episode of acute cardiac decompensation, requiring admission to the intensive care unit and initiation of inotropic support with Dobutamine (doses starting from 5 mcg/kg/min). Despite inotropic therapy, the severe biventricular systolic dysfunction persisted, and her overall clinical condition progressively deteriorated.

She was then referred to another pediatric cardiology clinic, Ospedale Pediatrico Bambino Gesu, Rome, Italy, for further management. Upon admission to this center, histopathological examination was performed and showed no evidence of inflammation and did not meet the histopathological criteria for acute, borderline, or chronic myocarditis. Specifically, the biopsy showed mild interstitial fibrosis and marked endocardial fibroelastotic thickening, without inflammatory infiltrates fulfilling the accepted quantitative diagnostic thresholds for myocarditis at the time of the biopsy. These alterations were consistent with the dilated cardiomyopathy phenotype. During her hospitalization in this clinic, given the patient’s INTERMACS profile 2 status, she underwent the implantation of an initially left Berlin Heart EXCOR, followed by the implantation of a right Berlin Heart EXCOR ventricular assist device, as a bridge-to-transplant therapy. After two months, due to lack of improvement of echocardiographic parameters under mechanical assistance and pharmacological treatment for heart failure, the patient met the criteria for inclusion on the heart transplant list. Mechanical circulatory support was maintained for more than 200 days, until transplantation. During this period, she presented multiple infections with Candida spp. and was treated with systemic antifungals (intravenous Fluconazol) and Berlin Heart exit-site infections with MRSA treated conservatively with systemic antibiotics (intravenous Cefepime).

The patient was the recipient of a cardiac transplant one year after the initial diagnosis. At that time, serological testing for Cytomegalovirus (CMV) and Toxoplasma spp. was negative.

Histopathological examination of the explanted heart confirmed extensive replacement fibrosis involving both ventricular free walls and the interventricular septum, together with diffuse left ventricular endocardial fibroelastosis. No histopathological features of active myocarditis, including lymphocytic, eosinophilic, or giant-cell myocarditis, were identified.

Immediately following transplantation, the patient developed severe right ventricular systolic dysfunction with preserved left ventricular contractility. The right ventricular systolic dysfunction gradually resolved over time. She remained under immunosuppressive treatment after the transplant. At first, treatment with systemic steroids and thymoglobulin was initiated, followed by chronic immunosuppressive therapy with Tacrolimus, Mycophenolate mofetil, and Prednisone. Chronic treatment with diuretics (oral Furosemide) and an ACE inhibitor (oral Enalapril) was maintained. One week postoperatively, the patient developed fever, and blood cultures were positive for Candida viswanathii. Treatment with systemic antifungal therapy was initiated, leading to microbiological clearance, with follow-up blood cultures remaining negative. One month after the transplant, cardiac catheterization and endomyocardial biopsy were performed, showing no signs of acute rejection and minimal focal interstitial fibrosis with rare CD3+ and CD20- lymphomonocytes and myocardial cells with no structural or microcellular alterations. In the first year following the transplant, serial echocardiographic evaluations demonstrated no structural cardiac abnormalities or transplant-related cardiac complications, with consistently preserved systolic and diastolic ventricular function. No rhythm or conduction disturbances were identified during electrocardiographic monitoring.

**Figure 3:**
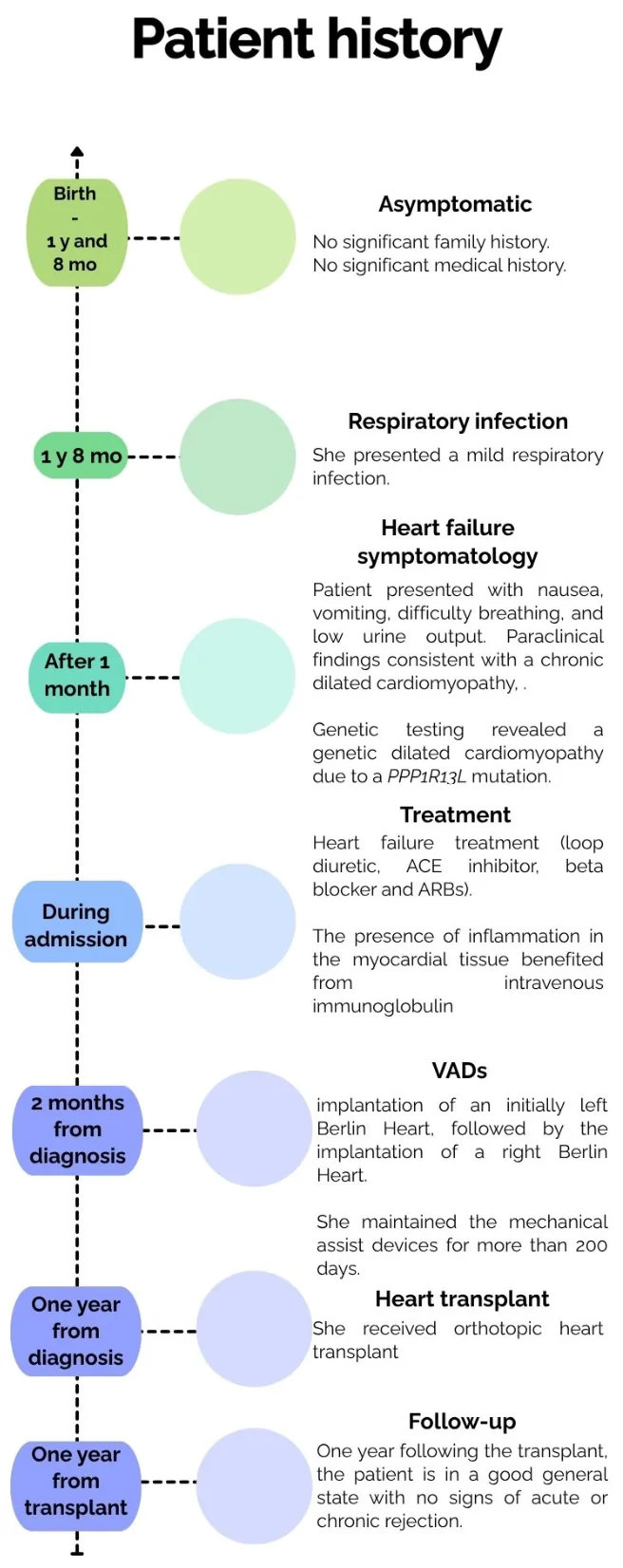
Chronology of the disease evolution and treatment strategy followed by the patient.

We present the timeline of the disease evolution in Figure 3.

## 2. Discussion

We have presented an exceptionally rare pediatric case of dilated cardiomyopathy (DCM) secondary to a novel or rare mutation in the *PPP1R13L* gene, culminating in a cardiocutaneous syndrome phenotype.

While primary cardiomyopathies represent only a single facet of the diverse clinical spectrum underlying pediatric heart failure, DCM carries substantial public health importance due to its profound morbidity and mortality rates [2,30]. Clinical management remains uniquely challenging, primarily due to the historical difficulties in establishing a definitive etiological diagnosis in most cases. To date, large-scale genomic investigations have predominantly focused on adult heart failure cohorts; consequently, genetic data regarding pediatric patients remain scarce and frequently limited by restricted sample sizes. Despite these historical data gaps, contemporary literature underscores the critical role of molecular screening in children. Notably, a landmark study published by Ware et al. in 2022 demonstrated that a pathogenic genetic variant can be identified in approximately 32% of pediatric DCM cases, yielding profound implications for targeted therapeutic management and family counseling [23].

Pathogenic variants within the *PPP1R13L* gene, localized to chromosome 19q13.32, have been increasingly associated with severe, frequently fatal cardiomyopathies, spanning both dilated and arrhythmogenic phenotypes. This gene encodes the inhibitor of apoptosis-stimulating protein of p53 (iASPP), a pivotal regulatory protein that maintains structural integrity by stabilizing desmosomal junctions between epidermal and myocardial cells. Beyond this structural role within cell-to-cell desmosomal complexes, a deficiency or loss of functional iASPP has been shown to unleash an exaggerated, uncoupled pro-inflammatory cascade within cardiomyocytes [24,31]. Translational models further support this dual-system pathophysiology: a study by Dedeić et al. in 2017 demonstrated that iASPP deletion in murine models reproduces human phenotypes, inducing rapidly progressive ventricular dysfunction alongside palmoplantar keratoderma and skin lesions when the protein was selectively knocked out in keratinocytes [32]. Given the extreme rarity of this condition and the broader scarcity of pediatric-specific genomic profiles, only a nominal number of clinical cases involving this specific pathogenic variant have been reported in the literature to date [23,24,25,33,34,35,36,37].

Having in mind the low number of cases presented in the literature, currently, a clinical trial involving patients with *PPP1R13L* mutations cannot be performed; thus, we present a table with the current reported cases of *PPP1R13L* gene mutations in pediatric patients (Table 1) [24,25,29,33,34,35,36,37].

In our 1-year-old patient, the initial clinical presentation strongly mimicked the acute decompensation of a silent, chronic cardiac substrate. Following a routine respiratory tract infection, the patient presented with possible acute myocarditis, which precipitated her acute heart failure symptoms. However, the profound severity of the baseline systolic dysfunction, juxtaposed against the total absence of cardiovascular symptoms prior to this inflammatory insult, strongly implied a preexisting, subclinical cardiomyopathy. This clinical discrepancy prompted an expansive etiological workup, ultimately revealing the rare *PPP1R13L* mutation. The patient’s rapidly progressive, treatment-refractory systolic failure closely mirrors the malignant phenotype currently described regarding this specific genetic variant. Furthermore, the co-occurrence of brittle nails and hair provided crucial physical evidence of an overlapping cardiocutaneous syndrome. However, owing to the small number of reported cases, the natural history and prognosis of cardiomyopathies associated with *PPP1R13L* mutations remain incompletely characterized.

Certain factors, including the etiology of dilated cardiomyopathy (DCM), have been identified as important prognostic modulators. In particular, the impact of genetic versus non-genetic etiologies on disease progression and clinical outcomes has been well established. Accordingly, the current guidelines of the European Society of Cardiology for the management of cardiomyopathies recommend early genetic testing not only for diagnostic purposes but also for prognostic stratification [27]. A recent review by Tetaj et al. discussed the role of genetic testing in guiding the management of DCM [38]. The authors concluded that the identification of high-risk genetic variants may have significant therapeutic implications, enabling more personalized risk assessment, surveillance, and treatment strategies.

The relation between myocarditis and genetic cardiomyopathy has been recently studied in the literature, as studies reviewing this relation have concluded that structural and molecular abnormalities could favor the persistence and progression of the inflammatory processes in acute myocarditis [39]. In a study published in 2002 by Xiong et al., the authors concluded that mutations in the dystrophin genes, as seen in neuromuscular diseases, can increase susceptibility to certain myocardial viral infections [40]. Additionally, certain studies have demonstrated a more severe clinical and paraclinical evolution in patients with concomitant myocarditis and genetic molecular alterations [41]. However, questions have been raised about the role of acute myocarditis in the development of dilated cardiomyopathy [39]. A study published in 2025 by Lutokhina et al. tried to answer this question. The authors found that the inflammatory processes act as a contributing factor in the phenotypic expression of genetic cardiomyopathies. Moreover, they determined that not the viral presence itself is the trigger for the subsequent inflammation, but the genetic alterations can induce a pathological response of the immune system as well [42]. That seems to be the case in the *PPP1R13L* mutation as well, as the loss of functional iASPP associated with *PPP1R13L* variants promotes a hyper-inflammatory myocardial state; the development of myocardial inflammation following a standard viral respiratory trigger in this patient may be directly explained by this underlying genetic vulnerability.

This was not the case for our patient. Despite the biological modifications (high CK-MB and troponin levels, with no biological inflammatory syndrome), the CMR aspect of early gadolinium enhancement suggestive of inflammation and the endomyocardial biopsy showed no signs of inflammation, either acute or chronic, thus excluding an acute myocarditis or an inflammatory myocardial process.

Intravenous immunoglobulin was still administered at that time due to the presence of the alterations in the myocardial tissue described by the CMR, as intravenous immunoglobulin has anti-inflammatory properties and can act as an immunomodulator. There have been cases in the literature where intravenous immunoglobulin has been used in other etiologies of dilated cardiomyopathy aside from acute myocarditis, such as systemic lupus erythematosus [43].

Another particularity of this case is represented by the lack of consanguinity, as most of the reported cases involve children from related parents (Table 1) [25,33,34,36,40]. Having in mind the fact that all the patients presenting DCM had a homozygous mutation of the *PPP1R13L* gene, parents might be carriers of a heterozygous mutation in this gene (Table 1).

As stated previously, this specific genetic mutation usually determines arrhythmogenic cardiomyopathy and ectodermal abnormalities. However, patients reported in the literature having this genetic mutation, including the present case, mostly present with a dilated cardiomyopathy phenotype.

Moreover, the variant described in our patient, to the best of our knowledge, has not yet been described in the literature. Additionally, this particular variant has not been described in population databases such as ExAC, genomAD, 1000G, or the ClinVar mutation database.

This case brings an important overview of the clinical course and prognosis of a dilated cardiomyopathy caused by a *PPP1R13L* mutation. While the majority of the cases presented in the literature have a negative outcome in the absence of heart transplant, our case presents a favorable clinical and paraclinical evolution following an orthotopic transplant.

## 3. Conclusions

Patients diagnosed with underlying genetic DCM, particularly those harboring *PPP1R13L* mutations, carry an exceedingly poor prognosis in the absence of orthotopic heart transplantation. Existent case reports confirm that disruptions in this gene predictably yield highly aggressive, life-threatening cardiomyopathies. This malignant trajectory was similarly observed in our patient, whose progressive hemodynamic decline required advanced mechanical circulatory support with biventricular assist devices (BiVAD) as a bridge to transplantation. However, no definitive conclusions can yet be drawn regarding the evolution or prognosis of cardiomyopathies associated with *PPP1R13L* mutations. Following successful orthotopic heart transplantation, the patient’s long-term clinical course has remained highly favorable. This case highlights the critical importance of early genetic testing in pediatric heart failure to unmask rare molecular etiologies, guide mechanical intervention timelines, and optimize transplant outcomes. The correlation between the clinical aspects and paraclinical investigations is essential to determine the etiology, pathophysiology, and disease evolution, as well as for guiding individualized patient management.

## Figures and Tables

**Table 1:** Cases of *PPP1R13L* gene mutations reported in the literature; DCM, dilated cardiomyopathy, EF, ejection fraction, ACM, arrhythmogenic cardiomyopathy, HCM, hypertrophic cardiomyopathy, HTx, heart transplant.

Authors, Year	Number of Patients	Variant	Consangvinity	Sex	Age at Presentation	Cardiac	Ectodermal Abnormalities	Outcome
Robinson et al., 2020 [24]	Child 1	c.2486_2487delinsTC c1610 del compound heterozygous	No	M	2 y 6 mo	DCM	Hair; nail	Death
	Child 2	c.736_764del homozygous	No	M	2 y	DCM	Nail	Death
	Child 3	c.21657A>C homozygous	Yes	M	9 y	DCM	None	Alive HTx
	Child 4	c.1537del c.1219C>T compound heterozygous	No	M	2 y	None	Hair; skin	Alive HTx
	Child 5	c.2396G>C homozygous	Yes	F	5 mo	DCM	None	Alive HTx
Tulbah et al., 2023 [33]	Child 1	c.988_989insC homozygous	Yes	F	2 y	DCM	Hair	Death
	Child 2	Not mentioned	Yes	F	2 y	DCM	Hair	Death
	Child 3	Not mentioned	Yes	M	33 mo	DCM	Hair	Death
	Child 4	Not mentioned	Yes	F	Birth	DCM	Hair; glaucoma	Death
	Child 5	c.988_989insC homozygous	Yes	F	2 y	DCM	Hair; glaucoma	Death
	Child 6	c.988_989insC homozygous	Yes	F	Birth	DCM	Hair; glaucoma	Alive
Acharya et al., 2026 [37]	Child 1	c.(2250_2249-1)(2448+1_2449-1) homozygous	No	M	5 y	DCM	Skin	Alive
Kalayinia et al., 2022 [25]	Child 1	c.580C>T homozygous	Yes	M	6 mo	ACM	No	Alive
Henry et al., 2022 [35]	Child 1	c.1068dup homozygous	Yes	F	2 y 10 mo	DCM	Hair	Alive
Kurt et al., 2025 [34]	Child 1	c.2368_2375dup	Yes	M	4 y	DCM	Hair	Death
Coudert et al., 2024 [36]	Child 1	c.1871_1872delGC	No	F	6 y	HCM	Hair	Death

## Data Availability

The data presented in this study are available upon request from the corresponding author.
